# Label-free LC-MS/MS proteomics analyses reveal CLIC1 as a predictive biomarker for bladder cancer staging and prognosis

**DOI:** 10.3389/fonc.2022.1102392

**Published:** 2023-01-16

**Authors:** Weifeng Wang, Guankai Huang, Hansen Lin, Lei Ren, Liangmin Fu, Xiaopeng Mao

**Affiliations:** ^1^ Department of Urology, The First Affiliated Hospital of Sun Yat-sen University, Guangzhou, China; ^2^ Institute of Precision Medicine, The First Affiliated Hospital of Sun Yat-sen University, Guangzhou, China

**Keywords:** proteomics, biology-informed analysis, CLIC1, prognosis, bladder cancer

## Abstract

**Introduction:**

Bladder cancer (BC) is a significant carcinoma of the urinary system that has a high incidence of morbidity and death owing to the challenges in accurately identifying people with early-stage BC and the lack of effective treatment options for those with advanced BC. Thus, there is a need to define new markers of prognosis and prediction.

**Methods:**

In this study, we have performed a comprehensive proteomics experiment by label-free quantitative proteomics to compare the proteome changes in the serum of normal people and bladder cancer patients—the successful quantification of 2064 Quantifiable proteins in total. A quantitative analysis was conducted to determine the extent of changes in protein species' relative intensity and reproducibility. There were 43 upregulated proteins and 36 downregulated proteins discovered in non-muscle invasive bladder cancer and normal individuals. Sixty-four of these proteins were elevated, and 51 were downregulated in muscle-invasive and non-muscle-invasive bladder cancer, respectively. Functional roles of differentially expressed proteins were annotated using Gene Ontology (GO) and Clusters of Orthologous Groups of Proteins (COG). To analyze the functions and pathways enriched by differentially expressed proteins, GO enrichment analysis, protein domain analysis, and KEGG pathway analysis were performed. The proteome differences were examined and visualized using radar plots, heat maps, bubble plots, and Venn diagrams.

**Results:**

As a result of combining the Venn diagram with protein-protein interactions (PPIs), Chloride intracellular channel 1 (CLIC1) was identified as the primary protein. Using the Gene Set Cancer Analysis (GSCA) website, the influence of CLIC1 on immune infiltration was analyzed. A negative correlation between CD8 naive and CLIC1 levels was found. For validation, immunohistochemical (IHC), qPCR, and western blotting (WB) were performed.Further, we found that CLIC1 was associated with a poor prognosis of bladder cancer in survival analysis.

**Discussion:**

Our research screened CLIC1 as a tumor-promoting protein in bladder cancer for the first time using serum mass spectrometry. And CLIC1 associated with tumor stage, and immune infiltrate. The prognostic biomarker and therapeutic target CLIC1 may be new for bladder cancer patients.

## Introduction

Bladder cancer is the tenth most common cancer worldwide, with approximately 573,000 new cases and 213,000 fatalities in 2020. Men are more likely than women to experience it. As a result, the illness is more prevalent in males, who demonstrate it as their sixth most frequent cancer and their ninth primary cause of cancer mortality than in women (1). It is estimated that 81,180 new cases of bladder cancer will occur in the United States in 2022, and 170,100 people will die from bladder cancer (2). In recent years, there has been a slight decline in the number of new bladder cancer cases and deaths related to bladder cancer in women. Men’s mortality rates have remained steady while incidence rates have declined (3). It is estimated that the number of new cases and deaths of bladder cancer in China in 2022 will be 84,825 and 19,223, respectively (4). Bladder cancer (BC) is a heterogeneous disease classified into muscle-invasive and non-muscle-invasive diseases (5). Muscle-invasive bladder cancer (MIBC) will be identified in 25% of bladder cancer patients, whereas NMIBC will be identified in the majority (MIBC). Recurrences or metastases, nevertheless, are common to most patients (6, 7). Although trimodally therapy is used in place of radical cystectomy to enhance the quality of life for individuals with BC (8), the chosen course of therapy for primary MIBC is still radical cystectomy (9–11). Trimodality treatment consists of transurethral resection with chemoradiation. Despite optimal treatment with surgery and chemotherapy, only 60% of muscle-invasive bladder cancer patients are alive five years after diagnosis (12). Clinical trials with checkpoint inhibitors are currently underway, and preliminary reports are promising (13–15). The gold standard for diagnosing BC in clinical settings continues to be cystoscopy and subsequent histological examination of the resected tissue. However, these procedures are intrusive and pricey and could result in urinary tract infections (16). Therefore, understanding the pathophysiology of bladder cancer, as well as identifying early diagnostic and prognostic indicators, is crucial for bladder cancer diagnosis, therapy, and prognosis assessment (17).

In recent years, biomarkers related to pathogenesis have been identified using high-throughput omics techniques (18–20). Promising sources for developing proteomic methods have been identified as serum. Blood samples are a convenient noninvasive liquid biopsy option since they are a great source of circulating abnormal proteins (21). With the advancement of metabolomic analysis using liquid chromatography-mass spectrometry (LC-MS), numerous blood-based proteins with differently expressed levels have been discovered in a variety of diseases, including cancer (18, 22, 23).

Our study examined the difference in the proteins in the serum of persons with bladder cancer and healthy people using liquid chromatography-mass spectrometry tandem analytic methods. After that, bioinformatics methods and technologies are used to obtain critical proteins for the functional analysis of the carcinogenesis and prognosis of bladder cancer. Additionally, we investigated the relationship between CLIC1 and multiple kinds of infiltrating immune cells. Meanwhile, we aimed to explore whether CLIC1 protein is associated with clinical and pathological parameters and survival rates. According to these studies, CLIC1 may serve as a therapeutic target and a prognostic marker for cancer patients.

## Materials and methods

### Sample collection and protein preparation

The current study consisted of 30 serum samples, 50 pairs of bladder cancer patient samples utilized for immunohistochemistry, and 16 pairs of bladder cancer tissues and adjacent non-cancerous tissues. Thirty cases of serum, including muscle-invasive and non-muscle-invasive bladder cancer, bladder papilloma, and healthy individuals. Fifty patients with bladder cancer provided paraffin sections and clinical data for immunohistochemistry and prognostic analyses. Bladder tumor specimens and surrounding non-cancerous bladder tissues were examined histopathologically at the First Affiliated Hospital of Sun Yat-Sen University’s Department of Pathology Department. Before surgery, none of the patients received adjuvant therapy, radiation, or chemotherapy. Liquid nitrogen was consumed to freeze and preserve the tissues together with the matched nearby non-cancerous bladder tissues. The serum was maintained at -80°C. The present research received permission from Sun Yat-Sen University’s First Affiliated Hospital ethics committee (Guangzhou, Guangdong, China)[2022]574.

First, serum samples are taken from a freezer at -80°C, and the cellular waste from the serum sample is spun at 12,000 g for 10 minutes at four°C to remove it. After that, a fresh centrifuge tube was filled with the supernatant. Using the PierceTM Top 14 Abundant Protein Depletion Spin Columns Kit, the top 14 proteins with the highest abundance were eliminated (ThermoFisher Scientific). Lastly, the BCA kit measured the protein concentration by the manufacturer’s recommendations.

Equal amounts of protein should be extracted from each sample, and the volume should be set with the lysate at a constant level. We alkylated a protein solution with 11 mM iodoacetamide at room temperature in the dark for 15 min after reducing it with five mM dithiothreitol at 56°C for 30 min. Placing the alkylated sample in an ultrafiltration tube, centrifuging it at 12,000 g for 20 minutes at room temperature, replacing it three times with 8 M urea, then three times with replacement buffer approximately one to fifty (protease: protein, m/m), before centrifuging it three more times. For enzymatic hydrolysis to occur overnight, trypsin should be introduced. We centrifuged the samples at 12,000 g for 10 min. At room temperature, the peptides were extracted. The two peptide solutions were combined after extracting them once again using ultrapure water.

### LC-MS/MS analysis

The ionized peptides that had been separated operating a UHPLC system and then introduced into an NSI ion source for ionization were analyzed using an Orbitrap Exploris™ 480 mass spectrometer. High-resolution Orbitrap was used to identify and analyze the peptide precursor ions and their secondary fragments. The voltage adjustment for the ion source was set at 2.3 kV, and the compensation voltage for FAIMS was set at -70 and -45 volts, respectively. There is a scanning resolution of 60000 and a scanning range of 400 to 1200 m/z on the central mass spectrometer; The secondary mass spectrometer’s fixed starting point for its scanning range was set to 110 m/z, 30000 was chosen as the secondary scanning resolution, and the TurboTMT was turned off. The data-dependent scanning (DDA) program is employed in the data acquisition mode. The first 15 peptide precursor ions with the highest signal intensity after the first-level scan are chosen. They are successfully injected into the HCD collision cell for fragmentation with a fragmentation energy of 27. This is followed by the second-level sequence, also performed in turn. Mass Spectrometry. For tandem mass spectrometry scanning, the maximum injection time was set to 100 ms, the device threshold to 1E4 ions/s, the automatic gain control (AGC) to 75%, and the dynamic exclusion period to 30 s to prevent repeated scans for precursor ions. These options were selected to maximize the use of mass spectrometry.

### Data analysis and bioinformatics analysis

The secondary mass spectrometry data in this experiment were collected using Proteome Discoverer (v2.4.1.15). The false positive rate (FDR) caused by random matching is determined by operating an anti-library; retrieval parameter setting. The database is Homo sapiens 9606 PR 20201214. fasta (75777 sequences); Trypsin (Full) is selected as the enzyme digestion technique, and 2 missed cleavage sites are selected. The mass error tolerance for the primary precursor ion was set to 10 ppm, and the second fragment ion’s mass error tolerance was set to 0.02 Da. A 1% threshold was applied to all proteins, peptides, and FDRs discovered by PSM.

After data filtering, the search results had 13800 and 2064 unique peptides and quantifiable proteins, respectively. Most peptides had an amino acid range of seven to 20, which was consistent with the general principles of enzymatic digestion and mass spectrometry fragmentation. The mass spectrometry-identified distribution of peptide lengths complied with the standards for quality control. Differentially expressed proteins (DEPs) were defined as fold values > 1.5 or < 0.667 and FDR < 0.05. The UniProt-GOA database (http://www.ebi.ac.uk/GOA/) was used to create the Gene Ontology (GO) annotation proteome. The protein ID should be mapped to the GO IDs after being converted to the UniProt ID from the identified protein ID. If some discovered proteins didn’t obtain UniProt-GOA annotations, the GO function of the annotated protein would be determined by the InterProScan program implementing a method based on the alignment of protein sequences. Proteins were categorized into groups using Gene Ontology annotation based on their functions as molecules, cellular components, and biological processes. The GO enrichment considered biological process (BP), molecular function (MF), and cellular component (CC). The conserved region of a particular protein sequence and structure known as a protein domain can exist, function, and evolve independently from the rest of the protein chain. Each domain may frequently be independently stable and folded, developing a microscopic three-dimensional structure. Several structural domains may be discovered in many proteins. Proteins with various differential expression levels may completely consist of the same domain. Domains are the building blocks of molecular evolution, and they may be rearranged in many ways to produce proteins with various functions. Between around 25 amino acids and 500 amino acids, domain lengths vary. The shortest domains, like zinc fingers, are held together by disulfide bridges or metal ions. The calcium-binding EF hand domain of calmodulin is an example of a functional unit. A chimeric protein, which consists of two proteins with swapped domains, can be constructed through genetic engineering thanks to the fact that each domain is stable on its own. The functional descriptions of the identified proteins were annotated using InterProScan, a sequence analysis tool, and the InterPro domain database. InterPro is a database that combines numerous data regarding protein families, domains, and functional sites and makes it freely available to the public *via* Web-based interfaces and services (http://www.ebi.ac.uk/interpro/). The foundation of the database is a set of diagnostic models, or signatures, that enable searches of protein sequences against them to determine potential functions. Besides large-scale genome and meta-genome studies, InterPro can also characterize unique proteins.

KEGG links information about known molecular interaction networks, including information about genes and proteins produced by genome projects, information about biochemical substances and processes, and information about pathways and complexes (the “Pathway” database) (including compound and reaction databases). The “protein network” and the “chemical universe” comprise two distinct networks that make up these databases. There are ongoing initiatives to expand our understanding of KEGG, including gathering data on the ortholog clusters found in the KEGG Orthology database. Metabolism, Genetic Information Processing, Environmental Information Processing, Cellular Processes, Rat Diseases, and Drug Development are the key KEGG Pathways. Protein pathways were annotated by managing the KEGG (Kyoto Encyclopedia of Genes and Genomes) database. The first step is to annotate the protein’s KEGG database description using the KEGG online service tool KAAS. The annotation results are mapped into the KEGG pathway database by employing the KEGG mapper from the KEGG online service.

Eukaryotic organisms have complexly segmented their cells into membrane-bound compartments that serve various physiological roles. Extracellular space, the cytoplasm, the nucleus, the mitochondria, the Golgi apparatus, the peroxisome, the vacuole, the cytoskeleton, the nucleoplasm, the nucleolus, the nuclear matrix, and the ribosomes are some of the key components of eukaryotic cells. We used WoLF PSORT, a software tool for predicting subcellular localization. The prediction of eukaryotic sequences is currently possible with Wolfpsort, a simplified version of PSORT/PSORT II.

By implementing GO annotation, proteins were divided into three groups: biological process, cellular compartment, and molecular function. A two-tailed Fisher’s exact test was administered for each category to determine if the differentially expressed protein was enriched compared to all other detected proteins. A corrected p-value < 0.05 is regarded as significant for the GO. Enhancement of pathway analysis: A two-tailed Fisher’s exact test was performed to determine enriched pathways using the Encyclopedia of Genes and Genomes (KEGG) database to compare the enrichment of the differentially expressed protein against all other detected proteins. P-values of less than 0.05 were considered significant pathways. The KEGG website’s classification of these pathways into hierarchical groups. Enrichment of protein domain analysis: A two-tailed Fisher’s exact test was carried out to examine the enrichment of the differentially expressed protein against all identified proteins. For each category of proteins, the InterPro database was searched. This database provides functional analysis of protein sequences by grouping protein sequences into families and estimating the presence of domains and crucial locations. These protein domains were significant when they had a corrected p-value of less than 0.05. To further examine functional PPI networks, we also analyzed functional PPI networks. Protein-protein interactions were looked up in every differentially expressed protein database entry or sequence using the STRING database version 11.5.

Unique interactions between the proteins in the searched data set were chosen, avoiding candidates from outside the data set. PPI network analyses were performed *via* Cytoscape (http://www.cytoscape.org, version 3.1.1) and the STRING database (http://string-db.org, version 11.5). Venn analysis tool was used to visualize the target proteins. A comprehensive study of the immune infiltrates’ correlation of CLIC1 in BC patients is performed by consuming the Gene Set Cancer Analysis website (http://bioinfo.life.hust.edu.cn/GSCA/). Bladder Urothelial Carcinoma was selected when CLIC1 was entered into the website, and GSCA computed Spearman’s correlations and reported statistical significance. The link between gene mRNA expression and immune cell infiltrates is examined using Spearman correlation analysis in the Immune infiltration & mRNA expression module. ImmuCell is used to assess the infiltration of 24 immune cells, including Bcell, CD4_T, CD8_naive, Cytotoxic, DC, Etc.

### Cell culture

Human bladder cancer cells T24, 5637, J82, RT112, EJ, TCCSUP, UM-UC-3, and human bladder immortalized epithelium cell line (SV-HUC-1) were purchased from Procell (Procell Life Science& Technology Co., Ltd). SV-HUC-1 cells were cultured in Ham’s F-12K medium. MEM mixed with 10% FBS (Excell) was used to cultivate the UM-UC-3 and the J82 line. RPMI1640 (Invitrogen) mixed with 10% FBS (Excell) was used to cultivate T24, 5637, RT112, EJ, TCCSUP, and those cell lines were all cultured in a water-saturated atmosphere with 5% CO2 at 37°C.

### qPCR

The total cellular RNA was extracted using TRIzol (Invitrogen, USA) reagent based on the protocol provided by the manufacturer and used to synthesize cDNA with the PrimeScript RT reagent kit (EZBioscience, China). EZBioscience 2× SYBR Green qPCR Master Mix (EZBioscience) was used for the procedure. Primers for qPCR were as follows. CLIC1 forward (5′- ACCGCAGGTCGAATTGTTC-3′) and CLIC1 reverse (5′- ACGGTGGTAACATTGAAGGTG-3′); ACTB forward (5′- CATGTACGTTGCTATCCAGGC-3′) and ACTB reverse (5′-CTCCTTAATGTCACGCACGAT-3′). TSINGKE produced every primer (Beijing TSINGKE Biotech Co., Ltd., China). After real-time PCR was carried out using the QuantStudio 5 Real-Time PCR machine, data were examined using the QuantStudio Design and Analysis software. The 2–ΔΔCt technique was implemented to measure relative gene expression, and ACTB was employed to normalize the results.

### Western blotting assay

After bladder cancer cells were lysed using NP-40 lysis buffer, protein quantities were assessed using a BCA Protein Assay Kit (Beyotime, China).10% Tris-Tricine SDS-PAGE was used to resolve the proteins and transfer them to polyvinylidene difluoride (PVDF) membranes. After that, the membranes were treated at 4°C for an overnight period with primary antibodies (rabbit antibody to CLIC1; Proteintech; Cat No. 14545-1-AP; β-tubulin; Proteintech; Cat No. 10094-1-AP). The membrane was then incubated with goat anti-rabbit IgG HRP (Abcam) at room temperature for another hour. A western blot substrate kit (Tanon, Shanghai, China) was used to observe the bands on the membranes. ImageJ software (https://imagej.nih.gov/ij/) was operated to quantify protein levels in western blotting assays.

### Immunohistochemistry

The samples were collected between January 2016 and December 2017 from Sun Yat-sen University’s First Affiliated Hospital. Pathology verified all of the diagnoses. Gradual alcohol was used to hydrate serial portions after deparaffinized in xylene—3.5 minutes of high vapor pressure antigen retrieval in 1X EDTA buffer (pH 9.0). After the slide was cooled, 3% hydrogen peroxide was applied for 10 minutes to stop endogenous peroxidase from functioning. By incubating for 60 minutes at room temperature in a moist atmosphere, 5% bovine serum albumin (BSA) prevented non-specific binding sites. Next, the sections were treated with primary antibodies directed against CLIC1 (anti-CLIC1, Proteintech; Cat No. 14545-1-AP) overnight at four °C. Hematoxylin counterstained the sections, and diaminobenzidine (ZLI-9018; Zhongshan Jinqiao Biotechnology Co. Ltd., Beijing, China) chromogen developed the color. Hematoxylin was used to stain the slides for 30 seconds, after which they were dried in graded alcohol and xylene. Five views for each tissue section were selected at random. The “uncalibrated OD” function was then used to calibrate the sections and the average of five views was recorded for each slide. scored the slides operating the Quick Score method to determine CLIC1 status within the tumor (24). Scores for the staining were 0 for negative, 1 for weak, 2 for moderate, and 3 for strong. Another batch of tissue sections was treated in the same way and stained with CD8 antibodies ((anti-CD8, Proteintech; No. 66868-1-Ig). Analyses comparing immunohistochemistry results to patient survival data were conducted using Kaplan-Meier survival methods.

### Statistical analysis

Statistics were performed using the student t-test with two tails and a chi-square test. Data on rank were subjected to the Mann-Whitney test. For the survival analysis, the Kaplan-Meier method was implemented, and tests of significance were conducted using log rank. The definition of statistical significance was P value < 0.05.

## Results

### LC-MS/MS analysis and identification of DEPs

Ten individuals with muscle-invasive bladder cancer make up Group A. Ten patients with non-invasive bladder cancer make up Group B. Group C consists of six patients with bladder papilloma, while Group D is made up of four healthy individuals. The successful quantification of 2064 Quantifiable proteins and 13,800 Unique peptides in total. According to the basic principles based on enzymatic digestion and mass spectrometry fragmentation, the majority of the peptides were distributed in the range of 7 to 20 amino acids. The mass spectrometry-identified peptide length distribution complied with the quality control standards (**Figure S1**). The intensity of LFQ for each protein in several samples is displayed in the search results (the original Intensity value of the protein is corrected between samples.). By center-transforming the protein’s LFQ intensity (I) in many samples, the relative quantitative value (R) of each sample’s protein is determined. Where I represent the sample and j denotes the protein, the calculation formula is as follows:

Rij=Iij/Mean (Ij)

Technical repeat data was tested for reproducibility based on Pearson’s correlation coefficients (**Figure S2**). First, the samples to be compared were chosen to identify differential proteins. The fold change was calculated as the ratio of each protein’s mean relative quantitative values in repeated samples (FC). Calculate, for instance, the protein fold difference between sample group A and sample group B. Where R represents the relative quantitative value of the protein, I represent the sample, and k represents the protein, the calculation formula is as follows:

*FC_A/B,k_=Mean(R_ik_,i∈A)/Mean(;R_ik_,i/B)*

T-tests were used to determine the significance of the difference between the samples from the comparison group and the samples from the control group. The associated P value was determined as the significance index, using P< 0.05 as the default value. The T-test necessitates that the test data conform to the normal distribution. The relative quantitative value of the protein must undergo Log2 logarithmic transformation before the test. Following is the computation:

Pk=T.test(Log2(Rik,i∈A),Log2(Rik,i∈B))

**Figure 1:**
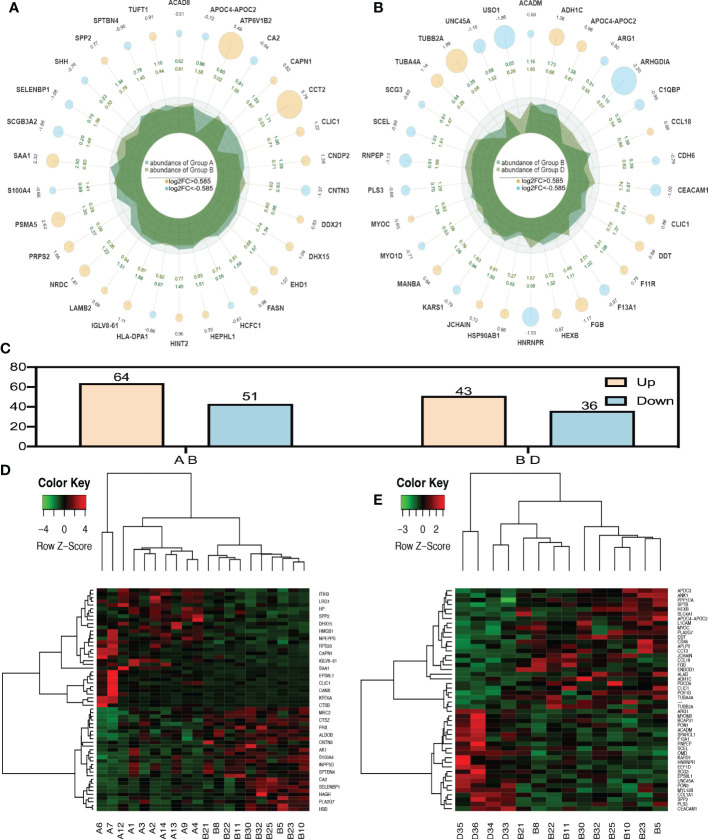
Radar plot and heatmap of differentially expressed proteins in various groups. **(A, D)** showed fold changes for proteins differentially expressed between group A and group B while **(B, E)** Are DEPs between group B and group D. In the Radar plot yellow circle and sky blue circle: up-regulated gene and down-regulated gene, and the circle size varies according to the log2 (FC) value; The second circle: the outer circle data represents the average expression of sample **(A)**; The inner circle data represents the average expression amount of sample **(B)**;Irregular shape in circle: abundance of expression of samples **(A, B)** on each axis. **(C)** was the number of all differential proteins. In the heatmap **(D, E)**, each row obtains protein. Each column is a sample/repeat, and different color represents different quantity expression.

When P value < 0.05, the differential expression change threshold of 1.5 or higher was used as the threshold for significant up-regulation, while the change threshold of less than 1/1.5 was used as the threshold for significant down-regulation. Based on **Figures 1A, C, D**, 115 differential proteins were identified between groups A and B, of which 64 proteins were upregulated and 51 were downregulated. According to LC-MS/MS, 36 were downregulated, and 43 were upregulated in group B compared to group D. (**Figures 1B, C, E**).

## Functional classification of DEPs

**Figure 2:**
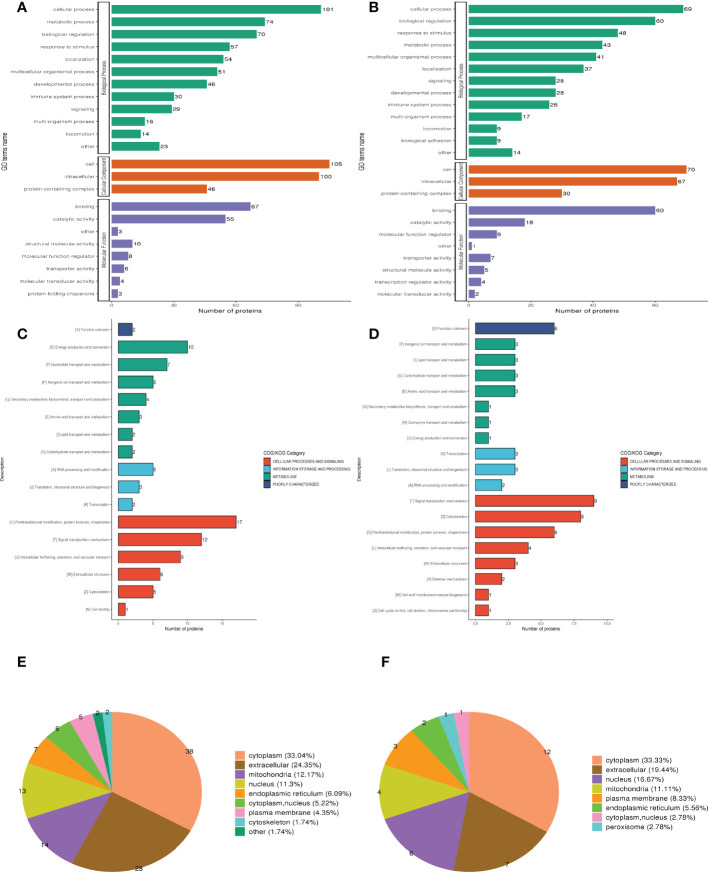
Clusters of Orthologous Groups of proteins (COG), Gene ontology (GO), and Subcellular localization annotation for functional classification. **(A, C, E)** Were the result of GO, COG, and Subcellular localization of DEPs between group A and group B, while **(B, D, F)** were the result of that between group B and group D. Different color represents various categories.

Three categories of GO functional annotation were performed: components of cells, biological processes, and molecular functions. Biological process ontologies were analyzed, and the primary GO terms that differentiated groups A and B were found in cellular process, metabolic process, and biological regulation (**Figure 2A**). The key GO terms that distinguished groups A and B in the analysis of cellular component ontology were discovered in “intracellular,” “cell,” and “ protein-containing complex. “ “Binding” and “catalytic activity” were identified as the dominant GO terms from molecular function ontology analysis that distinguished groups A and B. Moreover, the significant GO terms between groups B and D were the same as between groups A and B. Cellular process, response to stimulus, and biological regulation were identified as the three dominant GO terms that recognized groups B and D in the analysis of biological process ontologies (**Figure 2B**). In the analysis of cellular component ontology, the significant GO terms that identified groups B and D were identified in “intracellular,” “cell,” protein-containing complex, Binding, and catalytic activity for the molecular function category.

Clusters of Orthologous Protein Groups or COG. Orthologs obtain proteins from numerous species that have developed in vertical lineages (speciation), with the general understanding being that they carry out the same function as the original protein. The proteins that make up each COG are thought to have descended from an ancestor protein. Prokaryotes and eukaryotes are the two divisions within the COGs. In most cases, eukaryotes are referred to as KOG databases, and prokaryotes are referred to as COG databases. We performed COG/KOG functional categorization statistics on differentially expressed proteins by database alignment. Group A’s regulated functions compared to group B were categorized based on COG/KOG function (**Figure 2C**). **Figure 2D**, however, illustrates the regulated functional categorization of groups B and D.

The different membrane structures that proteins bind determine where they are located within eukaryotic tissue cells. We first utilized the WolF PSORT software to annotate the protein’s subcellular structure. Prokaryotic cells frequently lack the inner cell membrane, nuclear membrane-encapsulated nucleus, chromosomes, DNA strands that are not coiled and present in the cytoplasm in an accessible form, and the cytoplasmic membrane found in eukaryotic cells (such as mitochondria or chloroplasts). Based on this, we managed the PSORTb (v3.0) program to annotate the protein’s subcellular structure. Differentially expressed proteins between muscle-invasive and non-muscle-invasive bladder cancer were chiefly found in the cytoplasm (33.04%), extracellular (24.35%), and mitochondria (12.17%) of the cells (**Figure 2E**). The majority of the differential proteins in the non-muscle invasive bladder cancer and regular groups were located in the cytoplasm (30.38%), extracellular (24.05%), and nucleus (11.39%) (**Figure 2F**).

## Functional enrichment of DEPs

**Figure 3:**
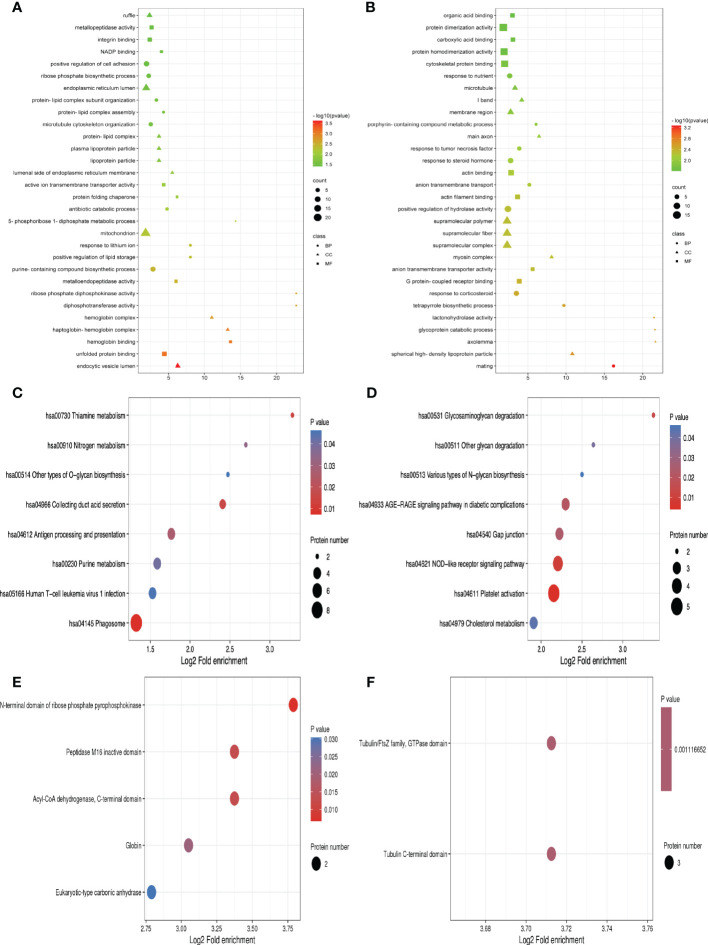
GO function, KEGG pathway, and protein domain enrichment analysis for all DEPs between groups. **(A, C, E)** Were the result between group A and group B, while **(B, D, F)** were those between group B and group D. Bubble size represents the number of DEPs; the enrichment test P value obtained by using Fisher exact test; varied color represents different P value.

For the differentially expressed proteins in each comparison group, we conducted enrichment analysis at three levels of GO classification, KEGG pathway, and protein domain (here, Fisher’s exact test was employed to determine the significant P value.). It aims to determine whether there is a discernible enrichment tendency in particular functional classes among the differentially expressed proteins. Bubble charts were used to show the functional categories and pathways where the differentially expressed proteins were substantially enriched (P<0.05). **Figure 3** displays the outcomes of the GO functional enrichment analyses. **Figure 3A** shows that group A and group B had enriched DEPs associated with “endocytic vesicle lumen,” “unfolded protein binding,” and “hemoglobin binding.” **Figure 3B** shows that group B and group D had enriched DEPs associated with “mating,” “spherical high-density lipoprotein particle,” and “axolemma.”

A network of known intermolecular interactions, including metabolic pathways, complexes, biological processes, Etc., are connected by KEGG. Metabolism, genetic information processing, environmental information processing, cellular activities, mortal illnesses, drug development, Etc., are the primary components of the KEGG pathway. The results of the KEGG analysis revealed that group A and group B’s DEPs were considerably enriched in “Phagosome,” “Collecting duct acid secretion,” and “Thiamine metabolism” (**Figure 3C**). Simultaneously, the DEPs in group B and group D were enriched in “Platelet activation,” “NOD-like receptor signaling pathway” and “Glycosaminoglycan degradation” (**Figure 3D**).

The building blocks of protein evolution are known as protein domains, which remain specific elements that often recur in many protein molecules and have similar sequences, structures, and activities. The length of a domain typically ranges from 25 to 500 amino acids. Protein domain enrichment analysis revealed that group A and group B differed in the “N-terminal domain of ribose phosphate pyrophosphokinase,” “Peptidase M16 inactive domain”, and “the Acyl-CoA dehydrogenase”(**Figure 3E**). Nevertheless, Tubulin C-terminal domain and Tubulin/FtsZ family, GTPase domain are the two protein domains that differ significantly between groups B and D (**Figure 3F**).

## PPI analysis and Venn analysis

**Figure 4:**
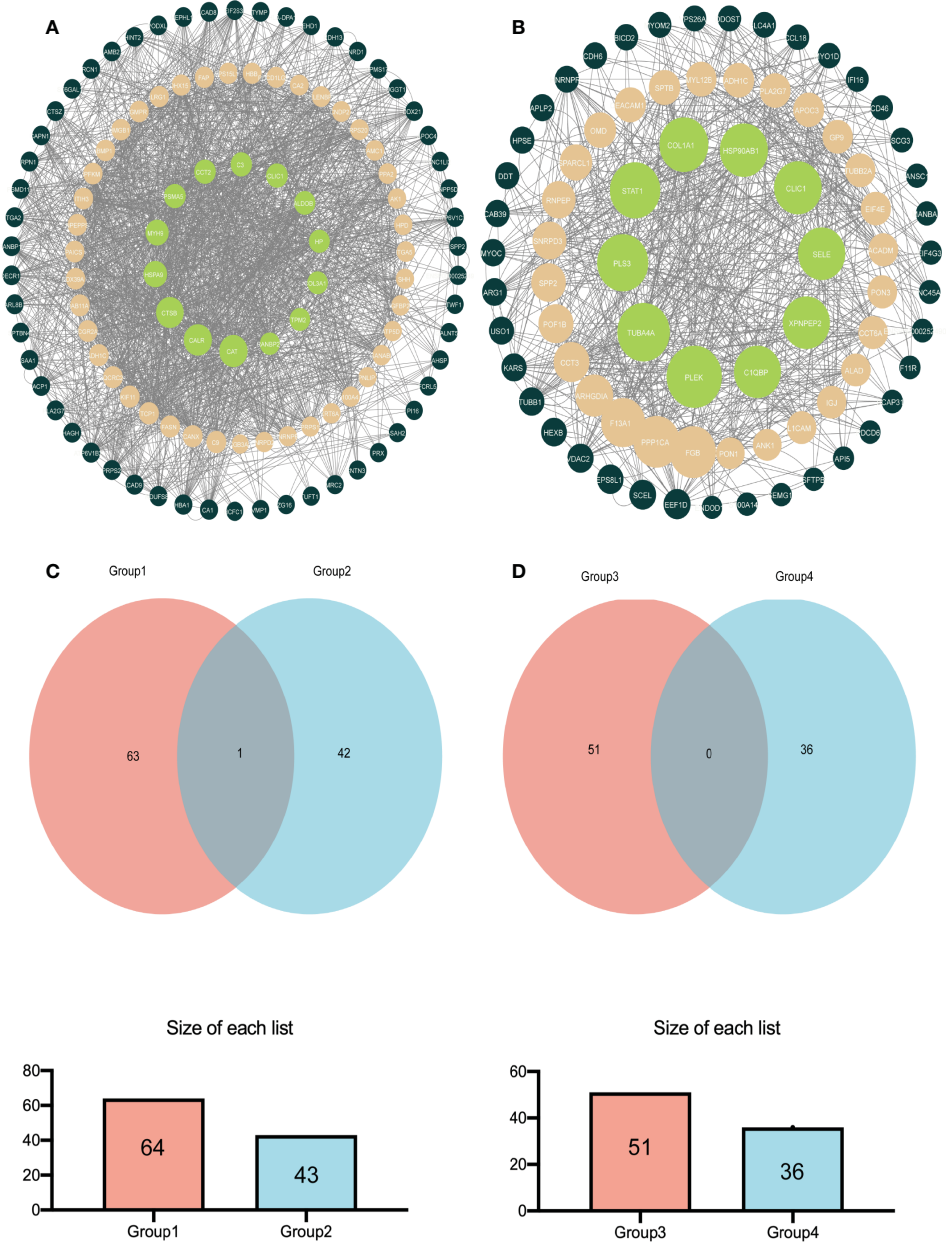
The relatively concentrated nets were obtained by PPl analysis and the Venn diagram of the DEPs between group A and group B vs group B and group D **(A)** Represent the key target network for DEPs in group A and group B **(B)** Represents the key target network for DEPs in group B and group D, the line represents the protein interaction recorded or predicted by STRING, and each node represents the key proteins recorded by Cytoscape. **(C)** Represents the common DEPs increased between group A and group B and increased between group B and group D **(D)** Represents the common decreased DEPs.

Analyzing the DEPs in various groups using STRING software in conjunction with Cytoscape software aided us in determining the molecular mechanisms that lead to bladder cancer. **Figure 4A** shows the main DEPs PPI network for group A and group B. In contrast, **Figure 4B** shows the leading DEPs PPI network for group B and group D. In addition, we employed Venn analysis to identify the essential proteins associated with bladder cancer (**Figures 4C, D**).

## Correlations between CLIC1 expressions and immune infiltration

**Figure 5:**
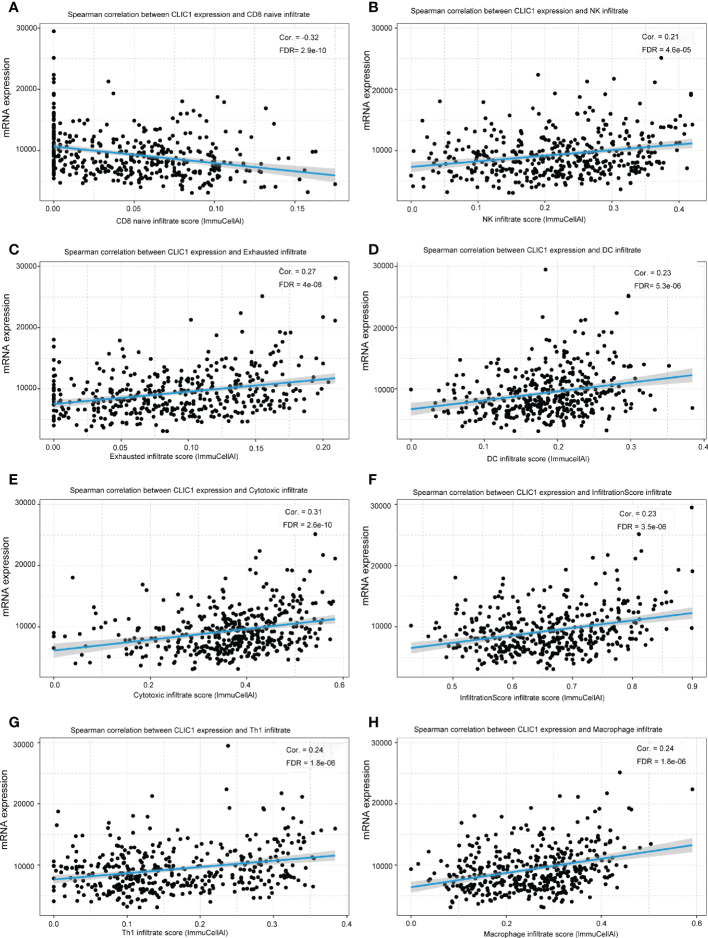
The relationship between CLIC1 and different immune cell infiltration levels. **(A)** Show that CLIC1 expression negatively correlates with CD8 naive infiltrate levels in bladder cancer. **(B-H)** A significant positive correlation is demonstrated.

The correlation between CLIC1 and specific immune infiltration cells, involving Central memory, Cytotoxic, T-helper 1 (Th1), InfiltrationScore, and regulatory T cells (Tregs), was analyzed by GSCA. *Significant correlation coefficients* were defined as cor >|0.2|and p< 0.05. Explicitly, CLIC1 revealed a significant negative correlation with CD8_naive, as well as a positive correlation with Cytotoxic, Dendritic cells (DC), exhausted T cells, InfiltrationScore, Macrophage, natural killer cell (NK), Th1 (**Figure 5**). However, no significant correlation between CLIC1 and the other immune infiltration cells.

### Expression of CLIC1 in the bladder cancer cells and tissues

**Figure 6:**
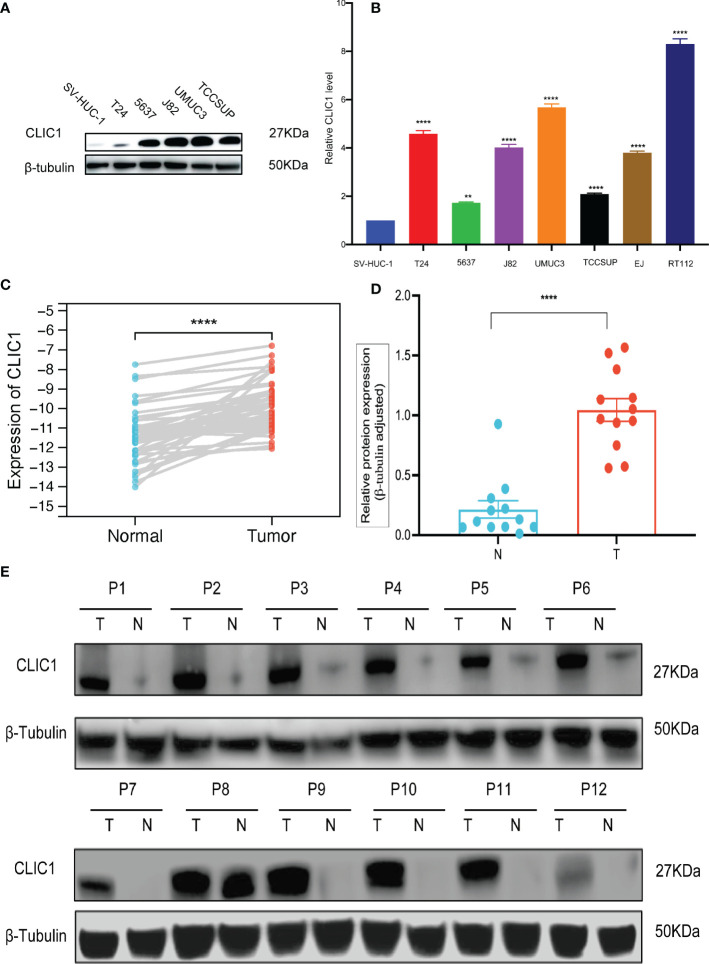
qPCR and WB analysis of CLIC1 expression in cell lines and tissues. **(A)** WB analysis of CLIC1 in cell lines. β-Tubulin WB was used to monitor protein loading. **(B)** CLIC1 mRNA expression level in SV-HUC-1 and bladder cancer cell lines. **(C)** CLIC1 mRNA expression level in paired primary bladder tumors and corresponding nontumor tissues. **(D, E)** WB analysis of CLIC1 and relative quantification. Gray value of relative expression levels of CLIC1 normalized to β-Tubulin. P < 0.05 was considered statistically significant. (**p ≤ 0.01, ****p ≤ 0.0001).

Cancerous bladder tissues and cell lines demonstrated more elevated levels of CLIC1 expression than normal bladder tissues and normal uroepithelium cells. Cell lines derived from bladder cancer possessed higher levels of CLIC1 expression than normal uroepithelium cells and were examined *via* RT-qPCR and western blotting. (**Figures 6A, B**). According to qPCR and Western blotting analyses, bladder tumor tissues contained significantly more CLIC1 protein than normal bladder tissues adjacent to the carcinoma. (**Figures 6C–E**).

### Immunohistochemical evaluation of CLIC1 and CD8

**Figure 7:**
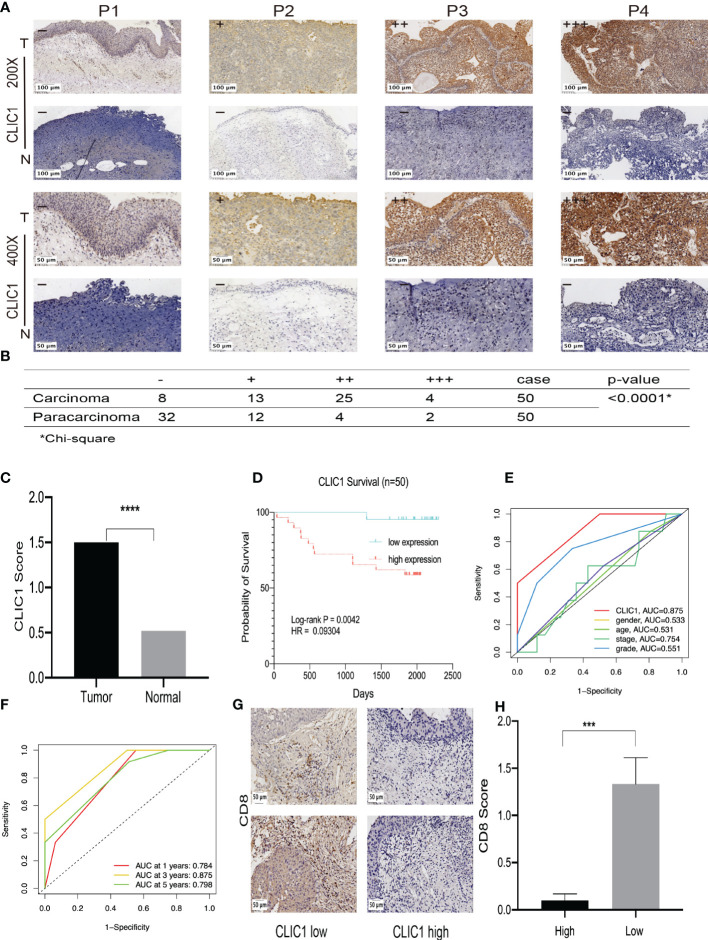
Immunohistochemistry of CLIC1 and CD8 expression and the Kaplan-Meier survival and ROC analysis of CLIC1 index in predicting the prognosis of BC. **(A)** Representative images of negative (−), weakly positive (+), moderately positive (++), and strongly positive (+++) CLIC1 staining by IHC analysis. **(B)** Statistical analysis of differing degrees of CLIC1 staining in bladder cancer tissues.**(C)** Expression levels (IHC score) of CLIC1 in bladder cancer tissues and paired adjacent paracarcinoma tissues. **(D)** Kaplan-Meier survival analysis based on CLIC1 expression levels. **(E)** Time ROC curves at 3 years of overall survival based on the CLIC1, age, gender, stage, and grade. **(F)** Time ROC curves at 1, 3, and 5 years of overall survival based on the CLIC1. **(G, H)** Immunohistochemistry for CD8. (*Chi-square, ***p≤0.001, **** p≤0.0001).

IHC was used to assess CLIC1 expression, and the results were based on how strongly the staining reaction occurred, as shown in **Figure 7A**. Among 50 bladder cancer tissue specimens, we found that eight patients had CLIC1- negative positives, 13 weak positives, 25 moderate positives, and four patients were strong positives. Among the tumor-adjacent normal bladder tissues, there were 32 cases of negative, 12 cases of weak positive, four cases of positive, and two cases of strong positive. In comparison with adjacent tissues, bladder cancer tissues demonstrated statistically significant differences (P<0.05) (**Figure 7B**). In the further step, we evaluated the association between CLIC1 and the clinicopathological features of the disease. CLIC1 expression was correlated with PT, and T2~T4 patients possessed higher levels of CLIC1 expression than Ta~T1 patients did. Age, gender, and pathological grade were unlinked in any way. (**Table 1**). CLIC1 expression was examined by detecting its protein expression and scoring the results on a 0 to 3 scale (**Figure 7C**). Kaplan-Meier survival curve and log-rank tests showed higher expression levels of CLIC1 were significantly correlated with poor patient outcomes based on 50 bladder cancer patients. (**Figure 7D**). Time-dependent receiver operating characteristic (time ROC) curves at three years of overall survival based on the CLIC1, age, gender, stage, and grade, and at 1, 3, and 5 years of overall survival based on the CLIC1(**Figures 7E, F**). CD8 expression was reduced in tumor tissues when CLIC1 was highly expressed but high when CLIC1 was expressed low (**Figures 7G, H**).

**Table 1:** Relationship between CLIC1 and clinical pathological characteristics in 50 patients with BC.

Variables		CLIC1 negative	CLIC1 weak positive	CLIC1 moderate positive	CLIC1 strong positive	Total	Z	P
Age	≤60	3	6	7	2	18	0.589	0.654
	>60	5	7	18	2	32		
Gender	male	7	11	20	3	41	0.642	0.568
	female	1	2	5	1	9		
pT	Ta~T1	8	7	15	0	30	2.444	0.015*
	T2~T4	0	6	10	4	20		
Grade	High	3	7	14	3	27	1.085	0.278
	Low	5	6	11	1	23		

*p≤0.05.

## Discussion

Potential biomarker research employs genomic, proteomic, and metabolomic techniques. Proteomics is the large-scale study of the features of proteins under different situations, including protein expression levels, post-translational modifications, protein interactions, Etc. Numerous serum proteomic studies have screened for biomarkers of various diseases (25–27). In this study, several cutting-edge technologies, including protein extraction, enzyme digestion, liquid chromatography-mass spectrometry tandem analysis, and bioinformatics analysis, were organically combined to conduct quantitative proteome research on samples.

Even though a growing body of research has shown CLIC1 may have an oncogenic role in human hepatocellular carcinoma and medulloblastoma, the protein’s function in BC has not yet been examined (28, 29). In this investigation, CLIC1 was highly expressed in bladder cancer tissues and circulated in the blood. As a result of the mass spectrometry results, CLIC1 was identified as a potential target associated with bladder cancer T stage. The GSCA analysis showed CLIC1 is negatively correlated with CD8 naive immune infiltration. We performed IHC staining on paraffin-embedded bladder tissues to determine CLIC1 and CD8 expression. CD8 T cells were discovered to be more infiltrated in bladder cancers from groups with faint CLIC1 expression. CLIC1 expression in bladder cancer tissue was 84% positive, of which 26% were weak positives (+), 50% were moderate positives (++), and 8% were strong positives (+++). The high expression of CLIC1 in bladder cancer tissue is consistent with previous studies (30). Ultimately, Kaplan-Meier survival curves showed that CLIC1-positive expression was closely related to shorter overall survival in BC patients. According to the findings, CLIC1 may be an oncogene and therapeutic target in BC.

A novel area of oncology research understands how ion channels contribute to the proliferation, invasion, migration, and metastasis of various cancer types (31–34). Ion channels, found in the plasma membrane, can monitor changes in the extracellular environment and react to them. As a result, they are essential for cell signaling and the development of cancer (35). Many aspects of cancer progression are facilitated by ion channels, including migration and metastasis (34, 36–38). Ion channel blockers are frequently prescribed to treat cardiovascular disease (39–41). Nevertheless, specific targeted therapy for cancer patients has yet to be unfound. The membrane remodeling, intracellular trafficking, vacuole formation, and actin rearrangement processes are all mediated by the six evolutionarily conserved members of the chloride intracellular channel (CLIC) family of proteins, CLIC1-CLIC6 (42–44). Chloride intracellular channel 1 (CLIC1), a protein from the CLIC family, is first found to be overexpressed in activated macrophages (45). In an ample range of tumors, CLIC1 is commonly overexpressed. CLIC1 expression has been linked to several malignancies’ poor prognoses and tumor growth, according to reports (28, 30, 46–49). Markedly, it has been reported that changes in CLIC1 are associated with poor prognoses in patients with gall cancer (50), pancreatic cancer (18, 48, 51), epithelial ovarian cancer (52), and hepatic cancers (53). CLIC1 induces cell-matrix adhesions for tumor metastasis, according to research by Peng, J.M. et al. (29). Qiu, Y. et al. found that CLIC1 knockout inhibits the invasion and migration of gastric cancer by upregulating AMOT-p130 expression (54). In cell proliferation, CLIC1 can auto-transmit from the cytoplasm to the plasma membrane without transport vesicles (55). Inhibiting CLIC1 activity may reduce or prevent distant metastases by preventing tumor proliferation and migration, which contribute to distant metastases (56). Previously, suppression of CLICl decreased proliferation and self-renewal properties in glioblastoma cells (57). However, CLIC1’s clinical significance in bladder cancer has yet to be determined. Therefore, we sought to uncover more about the function of CLIC1 in BC.

There is a vital role for tumor immune cell infiltration in the tumor immune microenvironment. Previously, it has been shown that overexpression of CLIC1 leads to CD8 cells undergoing apoptosis (58). The tumor microenvironment contains immune cells, which have proven valuable in predicting tumor prognosis (59). CD8T cells infiltrating tumors can be operated on to predict pancreatic cancer prognosis independently, and high CD8T cell infiltration is associated with prolonged survival (60). All these results indicate CLIC1 is associated with tumor invasion, migration, metastasis, and tumor immune microenvironment and could be an essential biomarker for diagnosis and therapeutic target.

In this study, some things still need to be improved. It is unknown if our findings apply to other groups because our subjects were Chinese. Next, Prognostic factors may equally be affected by differences in treatment regimens between patients. BC patients’ blood CLIC1 levels were undetected after treatment. The molecular mechanism of CLIC1 promoting BC occurrence, development, and metastasis was unstudied in cell or animal experiments. Other research, including more populations, is required. Additional research *in vivo* and *in vitro* validation would provide a more thorough understanding of CLIC1’s evolution from non-muscle invasive to muscle-invasive illness. Further research is needed to clarify the precise role CLIC1 plays in bladder cancer and investigate its potential contribution to bladder cancer prognostication.

## Conclusion

According to our knowledge, Our research screened CLIC1 as a tumor-promoting protein in bladder cancer for the first time using serum mass spectrometry. And CLIC1 associated with tumor stage, immune infiltrate, and prognosis. According to the previous results, CLIC1 may be a potential biomarker and therapeutic target for BC. More clinical research and biomolecular investigation would be required to supply more data for the subsequent in-depth studies.

## Data availability statement

The original contributions presented in the study are included in the article/**Supplementary Material**. Further datasets presented in this article are not readily available because they include patients' private clinical information. Requests to access the datasets should be directed to Weifeng Wang (wangwf26@mail2.sysu.edu.cn).

## Ethics statement

The studies involving human participants were reviewed and approved by Institutional Ethics Committee for Clinical Research and Animal Trials Ethical of the First Affiliated Hospital of Sun Yat-sen University. Written informed consent for participation was not required for this study in accordance with the national legislation and the institutional requirements.

## Author contributions

WFW and GKH: conceptualization, methodology, sofiware, data curation, formal analysis, and validation. HSL and LR : sample collection, pathology expertise, data analysis, and visualization. WFW drafted and edited the manuscript. LMF and XPM contributed equally to the correspondence work. LMF and XPM: Study coordination, guarantors, writing review. All authors contributed to the article and approved the submitted version.

## Supplementary material

Supplementary Figure 1 Control of Mass Spectrometry Data Quality. **(A)** Following a general guideline based on acid hydrolysis and mass spectrometry fragmentation patterns, most fractions were classified into 7–20 amino acids. **(B)** The coverage of the majority of proteins is less than 30%. (**C**) The detected proteins’ molecular weights were present at various stages and uniformly dispersed. **(D)** The majority of proteins correspond to several peptides.

Supplementary Figure 2 In Pearson’s correlation plot, the correlation values (r) between samples are visualized.
