# Corrigendum: Label-free LC-MS/MS proteomics analyses reveal CLIC1 as a predictive biomarker for bladder cancer staging and prognosis

**DOI:** 10.3389/fonc.2023.1216134

**Published:** 2024-01-09

**Authors:** Weifeng Wang, Guankai Huang, Hansen Lin, Lei Ren, Liangmin Fu, Xiaopeng Mao

**Affiliations:** ^1^ Department of Urology, The First Affiliated Hospital of Sun Yat-sen University, Guangzhou, China; ^2^ Institute of Precision Medicine, The First Affiliated Hospital of Sun Yat-sen University, Guangzhou, China

**Keywords:** proteomics, biology-informed analysis, CLIC_1_, prognosis, bladder cancer

**Incorrect Ethics No**

In the published article, there was an error in Ethics No. Instead of “[2022]385”, it should be “[2022]574”.

The authors apologize for this error and state that this does not change the scientific conclusions of the article in any way. The original article has been updated.

