# Supplementary figures and images for: Label-free LC-MS/MS proteomics analyses reveal CLIC1 as a predictive biomarker for bladder cancer staging and prognosis
Source: Front Oncol. 2023 Jan 16;12:1102392. doi: 10.3389/fonc.2022.1102392 (PMC9885092; doi:10.3389/fonc.2022.1102392)

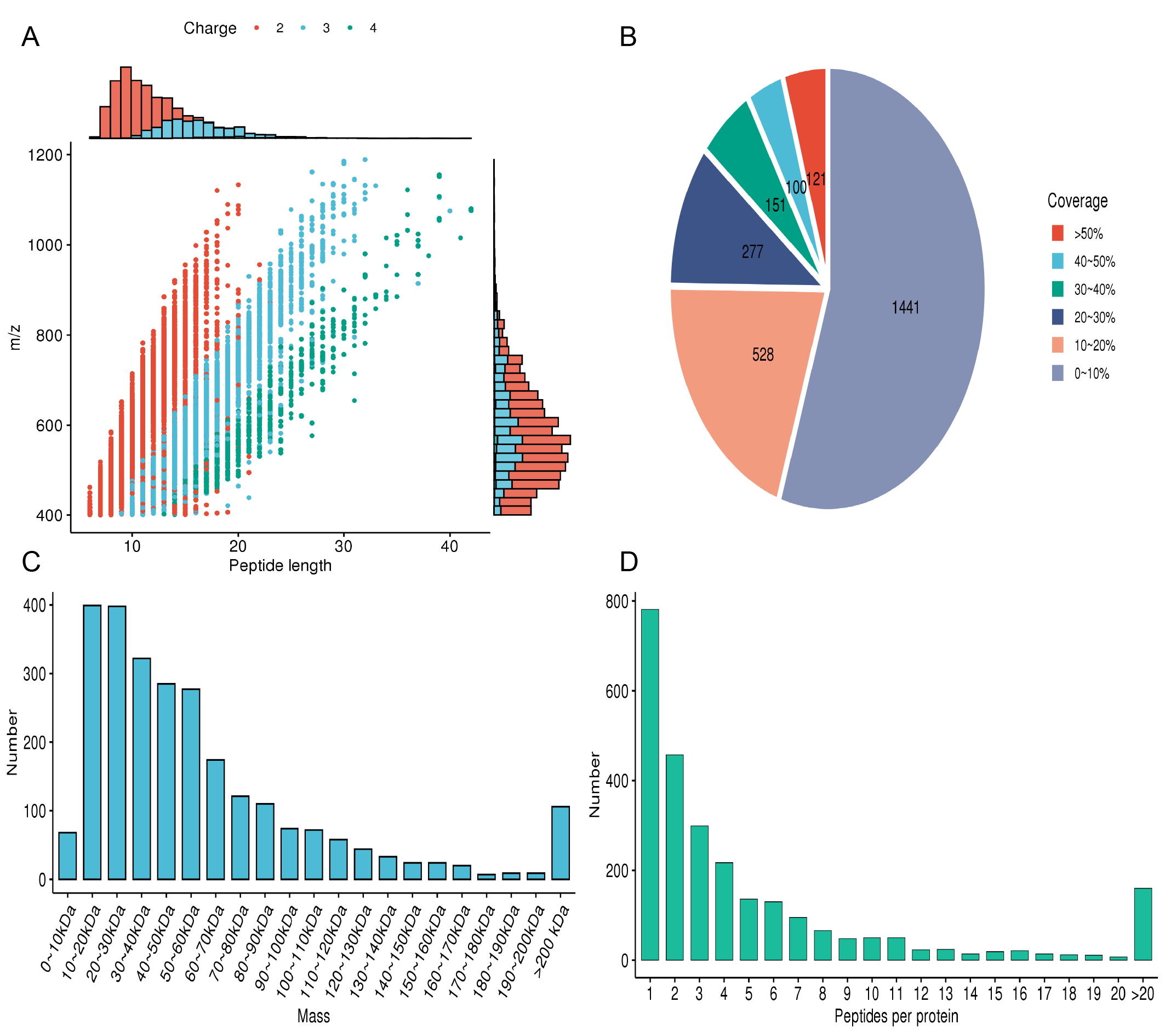

Supplement: Supplementary Figure 1 — Control of Mass Spectrometry Data Quality. (A) Following a general guideline based on acid hydrolysis and mass spectrometry fragmentation patterns, most fractions were classified into 7–20 amino acids. (B) The coverage of the majority of proteins is less than 30%. (C) The detected proteins’ molecular weights were present at various stages and uniformly dispersed. (D) The majority of proteins correspond to several peptides. [file Image_1.tif]

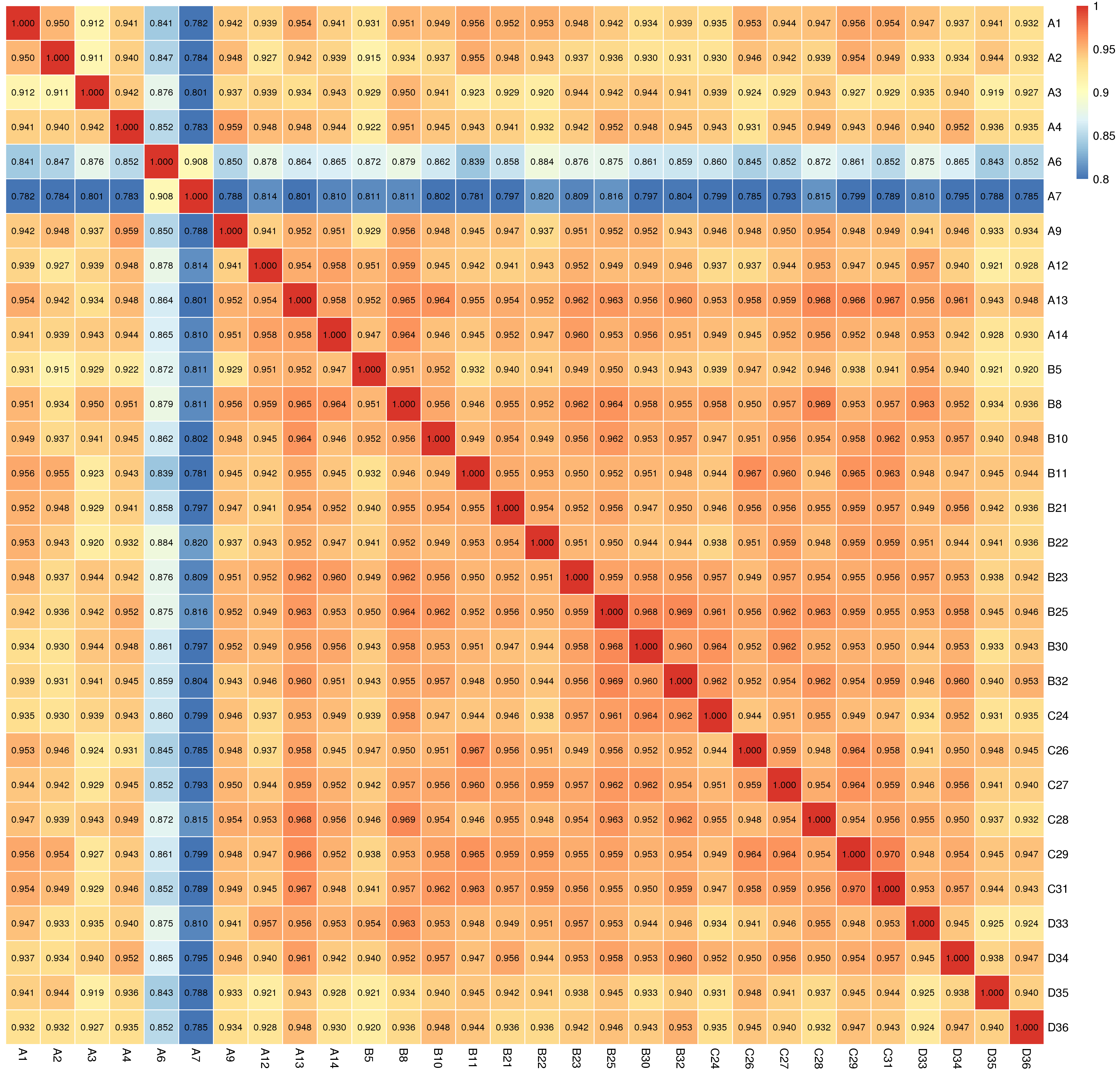

Supplement: Supplementary Figure 2 — In Pearson’s correlation plot, the correlation values (r) between samples are visualized. [file Image_2.tif]
