# Supplementary material for: Label-free LC-MS/MS proteomics analyses reveal CLIC1 as a predictive biomarker for bladder cancer staging and prognosis
Source: Front Oncol. 2023 Jan 16;12:1102392. doi: 10.3389/fonc.2022.1102392 (PMC9885092; doi:10.3389/fonc.2022.1102392)
Supplement: Supplementary file 4 [file DataSheet_2.zip › σÄƒσoïσ«₧Θ¬îμò░μì«/Fig-1.zip!/Fig-1/Fig1A,1B.docx]

Fig1A,1B

radar plots analysis was performed using the OmicShare tools,a free online platform for data analysis (<https://www.omicshare.com/tools>)
