# Supplementary material for: Label-free LC-MS/MS proteomics analyses reveal CLIC1 as a predictive biomarker for bladder cancer staging and prognosis
Source: Front Oncol. 2023 Jan 16;12:1102392. doi: 10.3389/fonc.2022.1102392 (PMC9885092; doi:10.3389/fonc.2022.1102392)
Supplement: Supplementary file 4 [file DataSheet_2.zip › σÄƒσoïσ«₧Θ¬îμò░μì«/Fig-1.zip!/Fig-1/Rplot01.pdf]

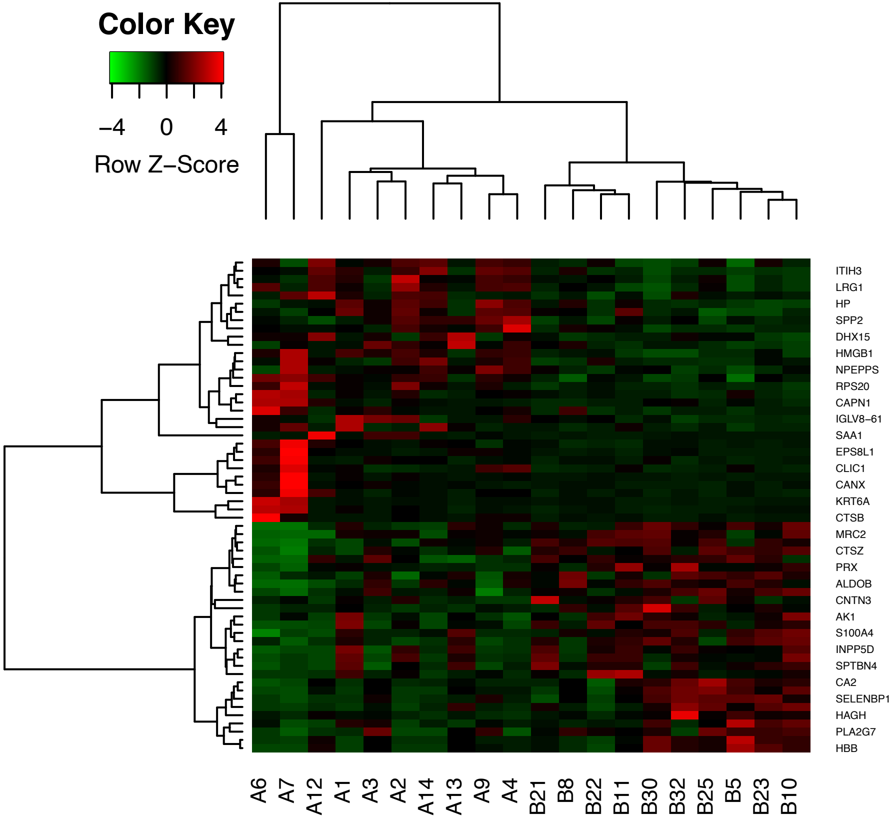

Color Key
−4 0 4
Row Z−Score
ITIH3
LRG1
HP
SPP2
DHX15
HMGB1
NPEPPS
RPS20
CAPN1
IGLV8−61
SAA1
EPS8L1
CLIC1
CANX
KRT6A
CTSB
MRC2
CTSZ
PRX
ALDOB
CNTN3
AK1
S100A4
INPP5D
SPTBN4
CA2
SELENBP1
HAGH
PLA2G7
HBB
A6
A7
A12
A1
A3
A2
A14
A13
A9
A4
B21
B8
B22
B11
B30
B32
B25
B5
B23
B10
