# Supplementary material for: Label-free LC-MS/MS proteomics analyses reveal CLIC1 as a predictive biomarker for bladder cancer staging and prognosis
Source: Front Oncol. 2023 Jan 16;12:1102392. doi: 10.3389/fonc.2022.1102392 (PMC9885092; doi:10.3389/fonc.2022.1102392)
Supplement: Supplementary file 4 [file DataSheet_2.zip › σÄƒσoïσ«₧Θ¬îμò░μì«/Fig-1.zip!/Fig-1/Rplot02.pdf]

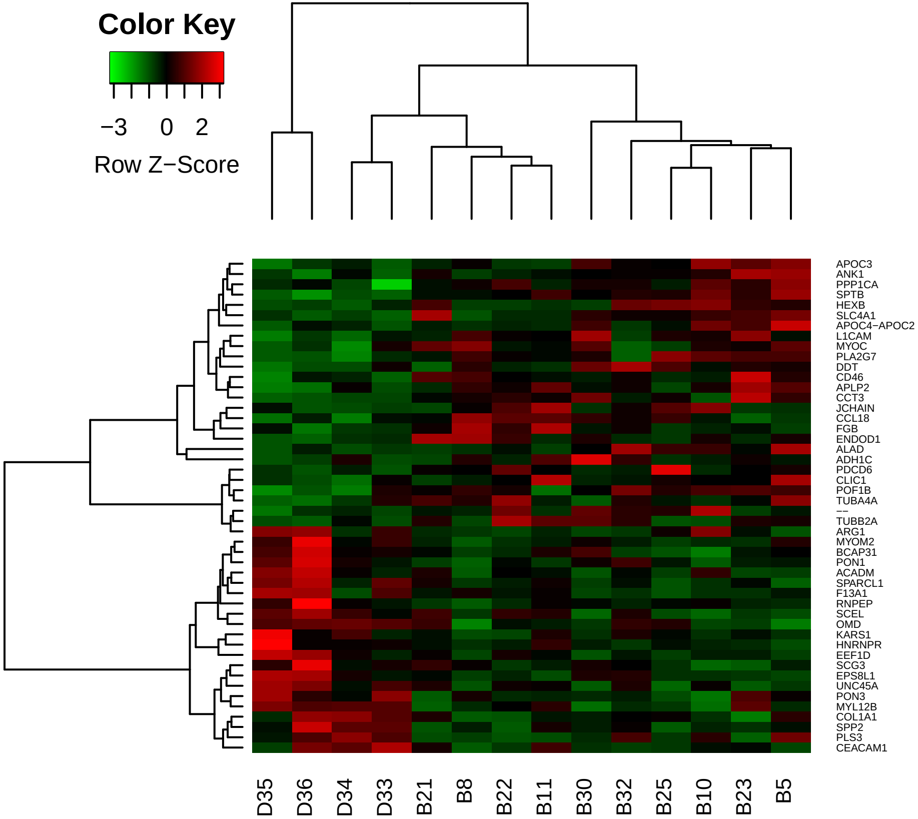

Color Key
−3
0
2
Row Z−Score
APOC3
ANK1
PPP1CA
SPTB
HEXB
SLC4A1
APOC4−APOC2
L1CAM
MYOC
PLA2G7
DDT
CD46
APLP2
CCT3
JCHAIN
CCL18
FGB
ENDOD1
ALAD
ADH1C
PDCD6
CLIC1
POF1B
TUBA4A
−−
TUBB2A
ARG1
MYOM2
BCAP31
PON1
ACADM
SPARCL1
F13A1
RNPEP
SCEL
OMD
KARS1
HNRNPR
EEF1D
SCG3
EPS8L1
UNC45A
PON3
MYL12B
COL1A1
SPP2
PLS3
CEACAM1
D35
D36
D34
D33
B21
B8
B22
B11
B30
B32
B25
B10
B23
B5
